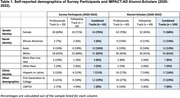# Long‐Term Assessment of Career Outcomes among US‐based Participants in a Novel AD/ADRD Clinical Trial Training Program: the IMPACT‐AD Experience

**DOI:** 10.1002/alz.095374

**Published:** 2025-01-09

**Authors:** Margaret D Mastrolorenzo, Maria C. Carrillo, Reisa A Sperling, Ronald C. Petersen, Paul S. Aisen, Heather M Snyder, Joshua D Grill, Rema Raman

**Affiliations:** ^1^ Alzheimer’s Therapeutic Research Institute, University of Southern California, San Diego, CA USA; ^2^ Alzheimer’s Association, Chicago, IL USA; ^3^ Brigham and Women’s Hospital and Department of Neurology, Massachusetts General Hospital, Harvard Medical School, Boston, MA USA; ^4^ Mayo Clinic, Rochester, MN USA; ^5^ Institute for Memory Impairments and Neurological Disorders, University of California, Irvine, Irvine, CA USA

## Abstract

**Background:**

The Institute on Methods and Protocols for Advancement of Clinical Trials in Alzheimer’s Disease and Alzheimer’s Disease Related Dementias (AD/ADRD) (IMPACT‐AD) is a novel multi‐disciplinary training program funded by the National Institute on Aging (NIA) and the Alzheimer’s Association for individuals seeking expertise in designing and conducting AD/ADRD trials. IMPACT‐AD offers a Professionals Track for investigators in a variety of trial roles and a Fellowship Track for future AD/ADRD clinical trial principal investigators.

**Method:**

To evaluate long‐term training outcomes, alumni‐scholars from the 2020‐2022 courses (n = 109) were surveyed via REDCap in January 2024. Measured constructs encompassed alumni‐scholar retention in AD/ADRD trials, promotions received, project funding, and the status of the protocol developed during the course, including whether it had been funded, conducted, and published (Fellowship Track). Alumni‐scholars rated the extent to which the course facilitated their achievements (not at all, somewhat, very much).

**Result:**

Of the 63 (58%) of alumni‐scholars who responded (48% Professional Track, 69% Fellowship Track), 44 (70%) were female. 21 (33%) identified as African American/Black, Asian, Multiracial or of another race; 7 (11%) self‐identified as Hispanic/Latino ethnicity. 5 **(**8%) identified as LGBTQ+. 12 (19%) indicated being first in their families to attend college.

Since program completion, 87% of survey participants maintained a career in ADRD clinical trials. A majority (63%) experienced a promotion or career advancement, with all indicating that IMPACT‐AD “somewhat” or “very much” contributed. Less than half of scholars (43%) had received project funding since IMPACT‐AD, but 74% of those had credited IMPACT‐AD at least somewhat for this achievement. Among the 34 Fellowship Track respondents, 15 (44%) indicated that the trial proposed at IMPACT‐AD had received funding, 14 (41%) reported its completion, and 5 (15%) had published its results. Nearly all respondents (%) reported assuming additional roles and responsibilities within their current title. In total, 92% of surveyed participants reported at least one positive change across all measured categories.

**Conclusion:**

IMPACT‐AD is effectively fulfilling its goals of training, nurturing, and diversifying a next generation of AD/ADRD trialists.